# Discovery of Highly Trimethoprim-Resistant DfrB Dihydrofolate Reductases in Diverse Environmental Settings Suggests an Evolutionary Advantage Unrelated to Antibiotic Resistance

**DOI:** 10.3390/antibiotics11121768

**Published:** 2022-12-07

**Authors:** Stella Cellier-Goetghebeur, Kiana Lafontaine, Claudèle Lemay-St-Denis, Princesse Tsamo, Alexis Bonneau-Burke, Janine N. Copp, Joelle N. Pelletier

**Affiliations:** 1 Department of Biochemistry and Molecular Medicine, Université de Montréal, Montréal, QC H3T 1J4, Canada; 2 The Québec Network for Research on Protein, Function, Engineering and Applications, Québec, QC G1V 0A6, Canada; 3 CGCC, Center in Green Chemistry and Catalysis, Montréal, QC H3A 0B8, Canada; 4 Chemistry Department, Université de Montréal, Montréal, QC H2V 0B3, Canada; 5 Michael Smith Laboratories, University of British Columbia, Vancouver, BC V6T 1Z4, Canada

**Keywords:** antibiotic resistance, type B dihydrofolate reductase, metagenomic database search, mobile genetic elements, multidrug resistance

## Abstract

Type B dihydrofolate reductases (DfrB) are intrinsically highly resistant to the widely used antibiotic trimethoprim, posing a threat to global public health. The ten known DfrB family members have been strongly associated with genetic material related to the application of antibiotics. Several *dfrB* genes were associated with multidrug resistance contexts and mobile genetic elements, integrated both in chromosomes and plasmids. However, little is known regarding their presence in other environments. Here, we investigated the presence of *dfrB* beyond the traditional areas of enquiry by conducting metagenomic database searches from environmental settings where antibiotics are not prevalent. Thirty putative DfrB homologues that share 62 to 95% identity with characterized DfrB were identified. Expression of ten representative homologues verified trimethoprim resistance in all and dihydrofolate reductase activity in most. Contrary to samples associated with the use of antibiotics, the newly identified *dfrB* were rarely associated with mobile genetic elements or antibiotic resistance genes. Instead, association with metabolic enzymes was observed, suggesting an evolutionary advantage unrelated to antibiotic resistance. Our results are consistent with the hypothesis that multiple *dfrB* exist in diverse environments from which *dfrB* were mobilized into the clinically relevant resistome. Our observations reinforce the need to closely monitor their progression.

## 1. Introduction

Trimethoprim (TMP) is a synthetic antibiotic that is intensively used worldwide as a result of its low cost and high effectiveness as a broad-spectrum treatment of bacterial infections [1]. TMP effectively inhibits bacterial dihydrofolate reductases (FolA) (e.g., K_i_ = 20 pM for *Escherichia coli* FolA)*,* abrogating the metabolically essential reduction of dihydrofolate (DHF) into tetrahydrofolate (THF) [2]. Shortly after the clinical introduction of TMP in the late 1960′s, TMP-resistant dihydrofolate reductases were identified in clinical samples [1,3]. In addition to TMP-resistant homologues of FolA (known as DfrA) [4], the evolutionarily unrelated type B dihydrofolate reductase (DfrB) DfrB1 was identified. Originally named R67 DHFR, DfrB1 circumvents the inhibition of FolA by TMP through catalysis of the dihydrofolate reduction in the presence of the antibiotic [3,5].

At the outset of this work, there were ten known DfrB family members (DfrB1–11; there is no DfrB8, [6,7,8,9,10,11,12]). All procure high TMP resistance in *E. coli* (MIC > 600 µg/mL; K_i_ ~0.38 to 1.3 mM), and most were originally identified in clinical samples [6,7,8,9,10,11,12]. The turnover rates of DfrB enzymes for dihydrofolate reduction (*k*_cat_ = 0.20–0.41 s^−1^) are at least 100-fold lower than bacterial FolA (e.g., *k*_cat_ = 230 s^−1^ for *E. coli* FolA); nonetheless, a low level of DfrB expression suffices to confer TMP resistance [13,14].

**Figure 1:**
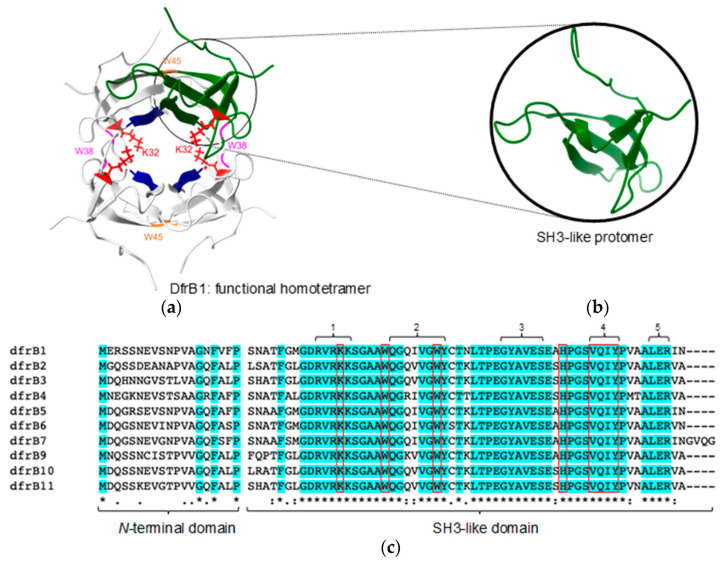
Structure of DfrB1 and sequence alignment of the known DfrB family members (75–95% sequence identity). (**a**) The functional, homotetrameric DfrB1 (PDB 1VIE) is constituted of four identical SH3-like protomers (one shown in green) that form the single, central active-site tunnel. The VQIY catalytic tetrad (V66, Q67, I68, and Y69; dark blue) and key residues K32 (red), W38 (magenta) and W45 (orange), are identified. (**b**) The DfrB1 protomer adopts an SH3-like fold. (**c**) Multiple sequence alignment of DfrB1–DfrB11 (there is no DfrB8) shows amino acid conservation, using standard annotation beneath the alignment. Conserved residues are highlighted in cyan. The poorly conserved *N*-terminal domain and the highly conserved SH3-like domain are identified. Functionally and structurally important residues are framed in red.

The absence of structural homology or sequence similarity with the ubiquitous FolA family of enzymes indicates that the DfrB family has a distinct evolutionary origin, where the dihydrofolate reductase (Dfr) activity is a result of functional convergence [4,15]. DfrB are homotetrameric enzymes constituted of four identical, 78-residue SH3-like protomers (Figure 1) [16]. Their highly conserved SH3-like domain (Figure 1 and Figure S1) includes the ‘VQIY’ (V66, Q67, I68 and Y69) catalytic tetrad that forms the single active-site cavity of the homotetramer [17], W38 and W45 for assembly of the functional tetramer [18], and K32 to establish electrostatic interactions with the substrates [4]. Although structural evidence has been obtained only for DfrB1 [16], conservation of all functionally and structurally essential residues as well as their comparable Dfr activity are consistent with adoption of the same functional tetrameric assembly in all DfrB family members [13].

We recently uncovered the mobility of *dfrB* genes found in pathogenic bacteria isolated from samples associated with human activity, such as clinical samples [11,12]. However, little is known about the presence or mobility of *dfrB* genes in environmental settings [19,20]. The small size of *dfrB* genes (~237 bp) and their unusual codon usage has impeded their discovery when using prokaryotic gene recognition tools, such as Prodigal, which discriminate against both these factors [12,21]. Recent bioinformatic developments facilitate identification of putative DfrB sequences. However, databases such as NCBI tend to be inherently biased towards clinical samples [22]. Metagenomic data can circumvent this limitation, as samples are collected from diverse environmental settings.

Our objective was to determine whether *dfrB* are identified predominantly in multidrug resistance contexts from samples associated with the use of antibiotics or whether they are also identified beyond those traditional areas of enquiry, without association to antibiotic resistance genes. To do so, we identified ten DfrB family members (DfrB12–DfrB21) from samples not associated with the use of antibiotics through a search of the JGI/IMG (Joint Genome Institute / Integrated Microbial Genomes) metagenomic database [23]. DfrB12–DfrB21 share 63% to 92% protein sequence identity with known DfrB family members (Figure S2). Expression in *E. coli* and characterization revealed that DfrB12–DfrB21 confer significant TMP resistance, and all but one display catalytic activities comparable to the known DfrB. Using similar search criteria, we identified ten further putative *dfrB* from the JGI/IMG database and ten more in NCBI to investigate their genomic context. Contrary to *dfrB* from samples associated with human activity, bioinformatic analyses revealed little association with mobility and multidrug resistance for environmental *dfrB* from samples not associated with the use of antibiotics. Identification of new *dfrB* in a variety of environments that are not directly associated with application of antibiotics confirms their widespread presence and suggests that the *dfrB* observed in the modern resistome may have originated from the mobilization of environmentally sourced *dfrB*.

## 2. Results and Discussion

### 2.1. Expansion of the DfrB Family

Following the recent identification and characterization of two new DfrB family members (DfrB10 and DfrB11) [12], our objective was to investigate whether further new DfrB homologues could be identified in environments that are less likely to be influenced by human activity. To this end, we queried the JGI/IMG metagenomic database and identified over 3000 *dfrB* gene homologues, from which ten sequences sharing 63 to 92% protein sequence identity were selected to be representative of sequence diversity (Figures S1 and S2). They were defined as DfrB12–DfrB21. High sequence identity of their SH3-like domain suggests that these DfrB12–DfrB21 should fold and tetramerize in a manner analogous to known DfrB enzymes, thus conferring high TMP resistance as a result of their Dfr activity. To investigate this, the minimal inhibitory concentration (MIC) of TMP for *E. coli* expressing DfrB12–DfrB21 was characterized, followed by determination of Dfr activity in *E. coli* lysate.

**Figure 2:**
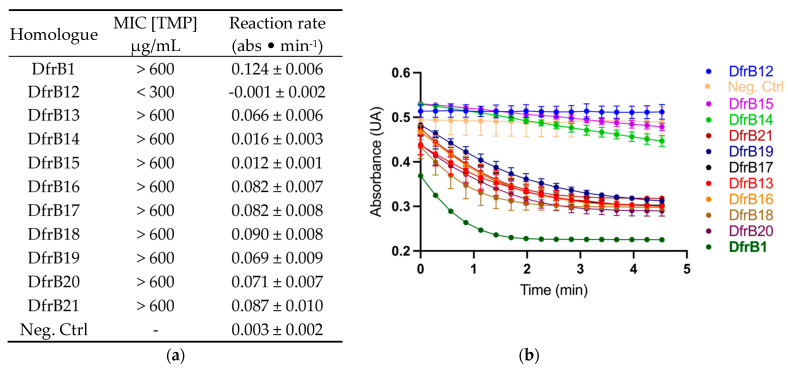
Newly identified DfrB homologues confer TMP resistance and possess Dfr activity. (**a**) Minimal inhibitory concentrations were determined on solid media with [TMP] ranging between 0–600 µg/mL. The reported MIC was the lowest TMP concentration where no bacterial growth was observed. The initial rates of reaction were calculated from panel B (*n* = 3, mean ± SD). The rate of the most active variants is underestimated, since the initial rate was not captured. (**b**) Dfr activity was determined in *E. coli* lysate, monitoring substrate consumption as a function of time (*n* = 3, mean ± SD). The negative control (Neg. Ctrl) is *E. coli* expressing the cTEM-19m β-lactamase instead of a DfrB.

Remarkably, all homologues except DfrB12 provided TMP resistance in *E. coli* comparable to that of DfrB1, up to the highest soluble concentration of TMP (600 µg/mL) (Figure 2A). Furthermore, activity was clearly observed in clarified lysate of *E. coli* for all DfrB homologues except DfrB12, which conferred resistance up to 150 µg/mL of TMP (Figure 2B). This apparent discrepancy results from little Dfr activity being required to sustain microbial proliferation, such that MIC assays are more sensitive than activity assays in crude lysate [12].

The lower TMP resistance and Dfr activity of DfrB12 relative to all other DfrB family members is most likely due to the Q67H substitution in the active-site VQIY tetrad (Figure S1). The Q67H mutation has been previously investigated: the mutation improves binding to both DHF and NADPH by 1–2 orders of magnitude compared to the native enzyme [24]. This favors the formation of the nonproductive DHF⋅DHF substrate or NADPH⋅NADPH cofactor complexes, resulting in an important decrease in activity. We note that this lower activity is sufficient to confer some TMP resistance. On the contrary, the 5- to 10-fold lower activity of DfrB14 and DfrB15 relative to DfrB1 is sufficient to confer the highest level of TMP resistance that we can measure. This work having been performed on crude lysate, we have not determined whether the reduced activity results from sequence variation outside of the conserved VQIY active site or other factors, such as reduced expression or stability.

These results confirm that DfrB12–DfrB21 constitute new DfrB family members. This demonstrates that identification of sequences sharing high sequence identity with *dfrB1–dfrB11,* including functionally and structurally important residues, is sufficient to identify new DfrB family members. This knowledge will facilitate robust identification of DfrB homologues in the future.

### 2.2. Genomic Context Analysis of DfrB12–21

The DNA sequences containing the newly identified *dfrB* genes originated from samples isolated from diverse environments not directly associated with the use of antibiotics (Table 1). Consistent with previous studies, *dfrB* genes were found in Proteobacteria [12]. As the identification of *dfrB* in diverse environments suggests their widespread presence, we investigated the mobility of the *dfrB12*–*dfrB21* genes by determining whether mobile genetic elements (MGEs, e.g., plasmids, transposons, or integrons) were present in their vicinity. Other antibiotic resistance genes (ARG) were also sought, because a major public health concern is transmission of ARGs associated with MGEs in pathogenic bacteria [25]. To allow comparison to *dfrB1*–*dfrB11*, mostly isolated from samples associated with human activity, we characterized the genomic context of *dfrB1*–*dfrB11* according to the same criteria (Figure S3).

First, sequences were classified as plasmidic or chromosomal using PlasForest and PlasFlow (Table S1) [26,27]. The resulting predictions obtained were often contradictory, such that it was difficult to conclude on their organization. The poor quality of predictions was expected, since the majority of analyzed contigs in that dataset were too short (<1 kbp) to allow for confident predictions [26].

The association of *dfrB* genes with integrons and transposon insertion sequences (IS) was investigated using IntegronFinder and ISFinder, respectively (Table 1) [28,29]. These tools rely on frequently updated databases as references and enable robust and precise identification of MGEs [28,29]. No contig contained transposon IS, but incomplete integrons (CALIN) were identified in the vicinity of *dfrB12*, *dfrB19*, and *dfrB20* [30]. Both *dfrB12* and *dfrB19* were integrated within a CALIN element, indicative of potential mobility of those two genes. The *dfrB20* gene was found outside of the CALIN identified in its genomic context, the longest obtained (nearly 10 kbp). Analysis of its content using BLASTP indicated 15 hypothetical or metabolism-associated proteins. Though not indicative of mobility of that *dfrB*, it demonstrates that genetic mobility occurred in the vicinity of the gene. Overall, this dataset contains evidence of genetic mobility in at most three among the *dfrB12–dfrB21* genes, in contrast with our earlier findings based on samples closely linked to human activity [12].

The Resistance Gene Identifier (RGI) tool from the Comprehensive Antibiotic Resistance Database (CARD) was used to assess the association of *dfrB12–dfrB21* with multidrug resistance (MDR, Table 1) [31]. In contrast to *dfrB1–dfrB11*, mostly identified in environments associated with the use of antibiotics and generally associated with MGE in a variety of MDR contexts (Table S3) [12], no ARGs were identified in this *dfrB12–dfrB21* dataset.

A clear limitation of the current dataset is the short length of the contigs (Table 1). Most genetic contexts were of insufficient length to allow identification of additional genetic features with confidence, indicating that analyses on longer contexts should be conducted.

### 2.3. The Broader DfrB Sequence Space Includes DfrB of Concern

To gain further information on the genetic context of the *dfrB* gene family, we identified ten further putative *dfrB* from a BLASTP search conducted in NCBI (referred to as putative *dfrB* B1–B10) and ten more from the above-described metagenomic JGI/IMG database search (referred to as putative *dfrB* C1–C10). We selected sequences with analyzable genomic context (>1 kbp) identified from environments that are not directly associated with the use of antibiotics (e.g., river sediments, soil), although some may be associated with human activity (e.g., polluted river sediment, wastewater). One sequence from a clinical sample (B5) was included as a basis for comparison. Although these new putative DfrB homologues were not functionally characterized, high sequence identity with *dfrB1–dfrB21* (63–92%) and conservation of all structurally and functionally important residues are consistent with their being functional DfrB homologues (Figure S3).

All sequences were predicted as chromosomal by PlasForest (Table 2), consistent with recent findings [12]. Complete integrons containing a *dfrB* gene (putative *dfrB* B1, B2, B5) and proximal transposases (putative *dfrB* B1, B5) were found only in contigs from samples collected in environments associated with human activity (Table 2). Strikingly, putative *dfrB* B1, B2, and B5 were also all associated with MDR (Table 2). Notably, previously known *dfrB* from clinical samples (*dfrB1–5*, *9–10*; Table S3) are all associated with clinical integrons and are in MDR contexts [12]. This is consistent with human-associated settings procuring higher TMP selective pressure, thus inducing mobilization of *dfrB* and acting as reservoirs for ARGs [32,33,34]. Our findings suggest that these MGEs have contributed to propagating *dfrB* from diverse sources into clinically relevant settings.

Additionally, putative *dfrB* B3 and B6 from water samples are from environments linked to human activity; they were found in MDR contexts but were not associated with MGEs (Table 2). This suggests vertical transmission or loss of mobility after acquisition of ARGs [35]. This was also the case for putative *dfrB* C3, which was isolated from soil in the Loxahatchee National Wildlife Refuge. The refuge accommodates a wide variety of recreational activities, although it is in a remote location; the relation of the sample to human activity is plausible but is not clear. Most ARGs found in the vicinity of putative *dfrB* B1–B3, B5-B6, and C3 are related to aminoglycoside resistance (*aadA16*, *AAC(6′)-IIa*, *ParS*, *aadA*, *baeS*), consistent with previous findings for *dfrB* of clinical origin [12]. Association with resistance to rifampin (*arr2*), chloramphenicol (*catB3*, *cmlA6*), beta-lactam (*OXA-21*), fosfomycin (*fosX*), polycationic antibiotics (*parS*), and macrolides (*mtrA*) was also noted. This demonstrates association of putative *dfrB* with MDR in environments linked to human activity beyond clinical contexts.

Conversely, indications of genetic mobility were found in the genomic context of putative *dfrB* B4 and B10 isolated from soil and putative *dfrB* C1 isolated from freshwater sediment, without association with MDR (Table 2). Strikingly, whereas analyses using CARD reveal no ARGs associated with those putative *dfrB*, BLASTP analyses of the integron content in the vicinity of putative *dfrB* C1 indicate the presence of ten proteins associated with metabolism and detoxification. This suggests the coevolution of putative *dfrB* C1 with metabolism- and defense-associated genes, rather than with antibiotic resistance genes. The remaining putative *dfrB* isolated from soil (C5, C6, C8–C10) and from water samples (B7, B8, C2, and C7) were not associated with MGEs or MDR (Table 2). These findings suggest that DfrB may confer an evolutionary advantage in environmental contexts that are not directly associated with the use of antibiotics.

All putative *dfrB* genes were isolated from Proteobacteria as for all known *dfrB*, most of which have been reported in clinical settings, often linked to ARGs and mobility (Table S3) [12]. Our findings highlight a new pattern: with few exceptions, the 12 putative *dfrB* genes identified in settings that are not associated with human activity were not associated with ARGs or mobility. Exceptions include the putative *dfrB* C4 linked with β-lactam resistance (*OmpA*) and an incomplete transposase (Table 2) and observation of *dfrB7* in a clinical integron (Table S3), despite both having been isolated from environmental sources. These examples could be the result of environmental contamination with clinically relevant pathogens [36]. Inversely, putative *dfrB* B9 was found in a wastewater sample but is not associated with MDR nor MGEs.

As the isolation sources and genomic contexts of these putative *dfrB* are heterogeneous, more information is needed to conclude on the influence of environment on mobility and prevalence of *dfrB*. Nonetheless, the association of *dfrB* from environmental sources with MGEs and/or ARGs demonstrates that the broader DfrB sequence space includes DfrB of concern and justifies the need to closely monitor them.

### 2.4. DfrB Genes with Similar Level of Mobility Share Closer Evolutionary Relationships

The identification of *dfrB* genes in various settings led us to investigate whether closer phylogenetic relationships exist between *dfrB* isolated from similar environments because of a higher likelihood of horizontal gene transfer [37]. To this end, the phylogeny of *dfrB1–dfrB21* and the 20 putative *dfrB* (B1–B10, C1–C10) was reconstructed using IQ-Tree [38].

**Figure 3:**
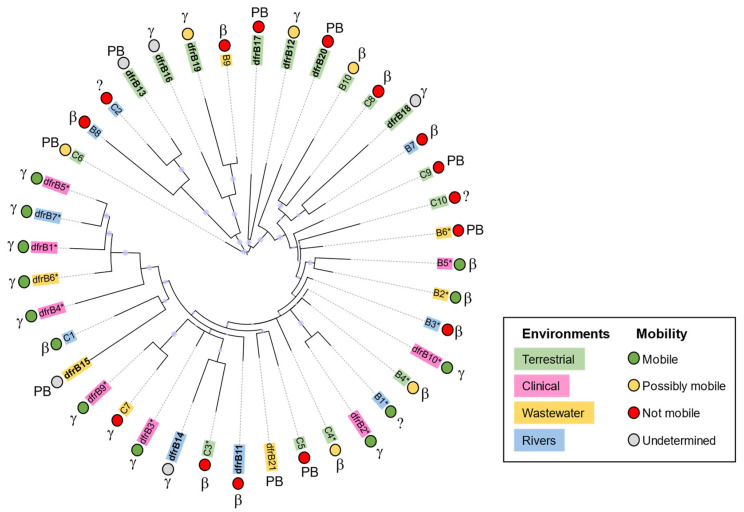
Phylogenetic tree of *dfrB1–dfrB21* and associated putative *dfrB* genes. Sequences are classified according to their source environment, predicted mobility, and taxonomy. Sequences were aligned using MAFFT; the tree was obtained using IQ-tree and visualized with iTOL. Bootstrap confidence levels are indicated by the size of the circle before each node. Information pertaining to source environment, taxonomy, and predicted mobility is reported in Table 2 and Table S3. The newly identified *dfrB12–dfrB21* are in bold. Sequences associated with MDR are marked with an asterisk (*). Taxonomy: “β” for β-Proteobacteria, “γ” for γ-Proteobacteria, “PB” for Proteobacteria; “?” indicates undetermined, as taxonomic information was not available in some cases.

These results highlight evolutionary proximity between sequences that have similar levels of mobility (Figure 3). For instance, most *dfrB* contained in integrons and associated with MDR (*dfrB1–dfrB5*, *dfrB9*, *dfrB10*, putative *dfrB* B1, *dfrB* B2, *dfrB* B5) share their closest ancestor with another integron-associated sequence.

These results also indicate evolutionary proximity between sequences from similar environments in the absence of indicators of mobility. For instance, pairs of *dfrB* from terrestrial samples (*dfrB16* and *dfrB19*; *dfrB12* and *dfrB17*; putative *dfrB* C8 and B10) share their most proximal common ancestor, although none hold clear markers of genetic mobility (Figure 3). This could suggest high conservation of the *dfrB* sequence owing to similar selection pressures from a similar environment and/or loss of mobility of an ancestral *dfrB*. In contrast, *dfrB* from samples isolated in aquatic or wastewater that are not associated with mobility are evenly distributed throughout the tree, suggesting that various evolutionary paths define their relationships.

Because all *dfrB* analyzed were found in Proteobacteria (Tables S2 and S3), it is difficult to distinguish events that are due to taxonomy from those due to horizontal gene transfer in our reconstructed phylogeny. Interestingly, *dfrB* from the same genus (e.g., *Rhodoferax sp*., putative *dfrB* B3 and B9) are not associated with mobility and do not share a close common ancestor. This could reflect different evolutionary pressures from different environments, as putative *dfrB* B3 was isolated from groundwater, whereas putative *dfrB* B9 was isolated from activated sludge. This also indicates that *dfrB* genes can exist in bacterial strains that are not typically associated with clinical settings [39], suggesting that DfrB enzymes could confer an evolutionary advantage in environmental contexts.

## 3. Conclusions

The results reported here demonstrate, for the first time, the widespread presence of *dfrB* in a diversity of environments. Most *dfrB* genes from samples not related to the use of antibiotics were not associated with markers of mobility nor of antibiotic resistance. Their association with metabolically relevant proteins and diverse evolutionary paths suggests that *dfrB* confer an evolutionary advantage unrelated to antibiotic resistance. Our results are consistent with the hypothesis that such environmentally sourced *dfrB* have been mobilized into the clinically relevant resistome, where they are associated with markers of mobility and antibiotic resistance. This work highlights the need to closely investigate and monitor their dissemination within the framework of developing therapeutic interventions to counter TMP resistance.

## 4. Materials and Methods

### 4.1. Identification of Putative dfrB Genes

Metagenomes deposited in the JGI/IMG database (https://img.jgi.doe.gov/) were queried using a Pfam search for “DHFR_2” on 8 May 2020. This returned a list of 2702 metagenomes, which were filtered for the Pfam keyword “pfam06442” [40]. This resulted in 3116 putative *dfrB* genes. Non-redundant sequences shorter than 100 amino acids approximating a full-length DfrB (78 amino acids) and starting with a methionine were filtered with CD-HIT [41]. Ten representative sequences sharing at most 95% protein sequence identity with any of the *dfrB1–dfrB11* genes were codon-optimized for *E. coli* and synthesized by Twist Bioscience (South San Francisco, CA, USA). These sequences had been subcloned into expression vector pET29 under control of the IPTG-inducible lac promoter.

Additional putative *dfrB* genes were identified by filtering the JGI/IMG metagenomic search results based on their nucleotide identity with *dfrb1–dfrB21* genes (<95%) and the length of their genomic context (>1 kbp). Complete coding sequences (234 nt = 78 amino acids) were prioritized. To identify further putative *dfrB* genes, the *dfrB1* sequence (Uniprot ID P00383) was used as a query for a BLASTP analysis using default parameters (10 January 2022). Results were filtered with CD-HIT to retain only sequences starting with a methionine and containing 78 amino acids while sharing 60–95% protein sequence identity with *dfrB1–dfrB21*.

### 4.2. Minimal Inhibitory Concentration (MIC)

MICs were determined in triplicates using the agar microdilution method. This was done as previously reported [12], with the following modifications. *E. coli* BL21(DE3) harboring one of the *dfrB*12–*dfrB*21 genes, *dfrB*1 (positive control), and TEM-1 β-lactamase variant cTEM-19m [42] (negative control) were propagated in 1 mL Luria-Bertani (LB) broth for 16–18 h at 37 °C with agitation at 230 rpm. LB–agar plates were prepared containing 0.25 mM IPTG (ThermoFisher). Plates were inoculated with 10^4^ colony-forming units per mL (CFU/mL) and incubated for 16–18 h at 37 °C. The lowest TMP concentration inhibiting visible bacterial growth was considered the MIC.

### 4.3. Dihydrofolate Reductase Activity Assays in E. coli Lysate

DfrB12–DfrB21, DfrB1 (positive control), and cTEM-19m (negative control) were overexpressed in *E. coli* BL21(DE3). An overnight (16–18 h) culture in LB (50 µg/mL kanamycin) was used to inoculate 1 mL ZYP-5052 autoinduction media [43] (for 1 L of media: 928 mL of ZY (1% tryptone, 0.5% yeast extract), 50 mL 20 × P (50 mM Na_2_HPO_4_, 50 mM KH_2_PO_4_, 25 mM (NH_4_)_2_SO_4_), 20 mL 50 × 5052 (0.5% glycerol, 0.05% glucose, 0.2% a-lactose), 2 mL MgSO_4_ (2 mM), and 0.2 mL 1000 × trace elements (50 mM FeCl_3_, 20 mM CaCl_2_, 10 mM MnCl_2_, 10 mM ZnSO_4_, 2 mM CoCl_2_, 2 mM CuCl_2_, 2 mM NiCl_2_, 2 mM Na_2_MoO_4_, 2 mM Na_2_SeO_3_, and 2 mM H_3_BO_3_) with 50 µg/mL kanamycin to obtain an initial OD_600nm_ of 0.1. The cultures were incubated at 37 °C, 230 rpm until the OD_600nm_ reached 0.7–1. Incubation was continued at 22 °C, 230 rpm for 16–18 h to allow protein expression. Cells were pelleted at 20,800× *g* for 30 min at 21 °C, and the pellets were stored at –72 °C until use. The pellets were thawed at room temperature and resuspended in 400 µL of lysis buffer (0.1 M KH_2_PO_4_-K_2_HPO_4_ (pH 8), 10 mM MgSO_4_ (Anachemia), 1 mM dithiothreitol (Fisher), 0.5 mg/mL lysozyme (MP Biomedicals), 0.4 U DNAse (Thermo), 1.5 mM benzamidine (Fisher), and 0.25 mM phenylmethylsulfonyl fluoride (Bioshop)) and kept for 2 h at RT with vigorous shaking. The lysates were centrifuged at 20,800× *g* for 30 min at 21 °C. The clarified lysates were used in subsequent assays.

DHF and NADPH in 50 mM KH_2_PO_4_-K_2_HPO_4_ (pH 7) were quantified by spectrophotometry (Cary 100 Bio UV-Visible, Agilent) using e^DHF^_282nm_ 28 400 M^−1^·cm^−1^ and e^NADPH^_340nm_ 6200 M^−1^·cm^−1^. In 96-well UV-transparent plates (Corning), 10 µL of clarified lysate was added to 100 µM NADPH and 100 µM DHF in 50 mM KH_2_PO_4_-K_2_HPO_4_ (pH 7) for a final volume of 100 µL. Enzyme activity was determined by monitoring the depletion of DHF/NADPH at 340 nm with a plate reader (Beckman Coulter DTX880) over 5 min. The initial rate of the reaction was determined by linear regression of the initial rate (first 20% of substrate consumption or the first minute) of depletion of both substrates (∆e_340nm_ 12 300 M^−1^·cm^−1^). Assays were carried out in triplicate.

### 4.4. Genomic Context Analysis

The contigs were classified as plasmidic or chromosomal using PlasForest [26] with the latest release of the NCBI database. Integrons were identified in contigs using IntegronFinder [28]. To perform this search, the local detection (--local-max) and search for promoter and attI sites (--promoter-attI) options were used. Transposon insertion sequences (IS) were identified in contigs using ISFinder BLASTN [29]. Antibiotic resistance genes were identified in contigs using the Resistance Gene Identifier (RGI) tool from the Comprehensive Antibiotic Resistance Database (CARD) [44].

### 4.5. Phylogenetic Tree

Amino acid sequences were aligned using MAFFT [45] with the default options. A phylogenetic tree was constructed using IQ-Tree [38] with the Ultrafast bootstrap analysis (1000 alignments, 1000 iterations, 0.99 minimum correlation coefficient). Branch support was determined using the SH-aLRT branch test (1000 replicates) and the Approximate Bayes test. The JTT+G4 substitution model was selected using the automatic model selection option. The resulting consensus tree was visualized using iTOL and rooted using the Midpoint root function [46].

## Figures and Tables

**Table 1:** Genomic context analysis of *dfrB12–21*.

Name and Position ^a^	Genomic Context Length (bp) ^b^	Environment ^a^	Host Strain ^a^	Integron ^c^	Insertion Sequences ^d^	Antibiotic Resistance Genes ^e^
*dfrB12* (317..553)	743	Soil, Arlington Agricultural Research Station	*E. coli*	CALIN (2..676)	None	None
*dfrB13* (10..246)	540	Soil, Arlington Agricultural Research Station	Proteobacteria	None	None	None
*dfrB14* (97..333)	450	Freshwater sediment, Lake Washington	*E. coli*	None	None	None
*dfrB15* (93..329)	562	Wastewater effluent	Proteobacteria	None	None	None
*dfrB16* (191..427)	596	*Populus* sp. Microbial communities, riparian zone	*E. hormaechei*	None	None	None
*dfrB17* (1065..1301)	1715	Desert sand, soil crust	Proteobacteria	None	None	None
*dfrB18* (11..247)	359	*Barbacenia macratha* root associated microbial communities	*E. hormaechei*	None	None	None
*dfrB19* (702..938)	1077	Fen, Stordalen Mire	*E. hormaechei*	CALIN (103..1001)	None	None
*dfrB20* (9332..9568)	9717	*Populus trichocarpa* microbial communities, riparian zone	Proteobacteria	CALIN (1..9097)	None	None
*dfrB21* (288..524)	625	Uranium-contaminated sediment slurry	Proteobacteria	None	None	None

^a^ Determined from available information in the JGI/IMG metagenomic database. ^b^ Number of base pairs (bp) in each contig containing a *dfrB*. ^c^ Complete and incomplete (CALIN) integrons were searched for with Integron Finder. Where applicable, the type of MGE identified and its position in the contig are indicated. ^d^ Searched for with ISFinder. ^e^ Searched for on the CARD database.

**Table 2:** Genomic context analyses of putative *dfrB* from BLASTP and JGI/IMG searches.

Name and Position ^a^	Genomic Context Length (bp) ^b^	Environment ^c^	Integron ^d^	Insertion Sequences ^e^	Organization ^f^	Antibiotic Resistance Genes ^g^
B1 (1..251)	1890	Polluted river sediment	Complete (3…1890)	Tn3 transposase (1051...1890)	Chromosomal	*arr2*
B2 (2467710..2467946)	4,664,715	Wastewater	Complete (2462823...2469276)	None	Chromosomal	*aadA16*, *catB3*, *OXA-21*, *AAC(6′)-IIa*
B3 (270150..270386)	341,798	Groundwater (48 m deep), Hainich Critical Zone Exploratory	None	None	Chromosomal	*FosX*, *ParS*, *mtrA*
B4 (1436..1672)	3039	Soil, Usan-dong village	CALIN (1...2856)	None	Chromosomal	None
B5 (1695128..1695367)	4,457,823	Clinical, human sample	Complete (1692032...1696644)	TnAs3 transposase (1679393...1683642)	Chromosomal	*aadA*, *cmlA6*
B6 (2901..3137)	14,187	Activated sludge, wastewater treatment plant	None	None	Chromosomal	*baeS*
B7 (175053..175289)	844,006	Groundwater (<100 m deep)	None	None	Chromosomal	None
B8 (802..1038)	4,496,947	River, hydroelectric dam	None	None	Chromosomal	None
B9 (1026..1262)	41,160	Activated sludge, wastewater treatment plant	None	None	Chromosomal	None
B10 (19507..19743)	82,085	Forest acidic soil	None	ISNCY transposase (13699...15117)	Chromosomal	None
C1 (3299..3535)	23,478	Freshwater, Lake Lanier	Complete (868...7457)	None	Chromosomal	None
C2 (498..734)	5709	Freshwater, selected watersheds (little to no prior anthropogenic activities)	None	None	Chromosomal	None
C3 (3371..3607)	4299	Soil, wildlife refuge	None	None	Chromosomal	*AAC(6′)-Iak*
C4 (3287..3523)	3806	Soil, Bohemian Forest Mountain range (1170–1200 m altitude)	None	ISCARN35 transposase (3725...3806)	Chromosomal	*OmpA*
C5 (1221..1457)	2462	Soil, coastal freshwater wetland	None	None	Chromosomal	None
C6 (1262..1498)	2430	Soil, coastal reserve	None	None	Chromosomal	None
C7 (1466..1702)	2288	Biofilm, wastewater treatment plant	None	None	Chromosomal	None
C8 (1246..1482)	2134	*Miscanthus* sp. rhizosphere	None	None	Chromosomal	None
C9 (511..747)	2075	*Populus trichocarpa* ectomycorrhiza	None	None	Chromosomal	None
C10 (694..930)	1938	Sugarcane root	None	None	Chromosomal	None

^a^ Sequences B1–B10 were retrieved from NCBI and C1–C10 from JGI/IMG. ^b^ Number of base pairs (bp) in genomic context. ^c^ Environmental source was determined using the available information on the NCBI or IMG/JGI database. ^d^ Complete and incomplete (CALIN) integrons within 5 kbp of a *dfrB* were searched for with IntegronFinder. Where applicable, the type of MGE identified and its position in the contig are indicated. ^e^ Searched for with ISFinder, within 5 kbp of a *dfrB*. ^f^ Organization as chromosomal or plasmidic was predicted with PlasForest using DNA contig sequences. ^g^ Searched for on the CARD database within 5 kbp of a *dfrB*.

## Data Availability

Data are contained within the article or in the supporting information. The accession numbers of analyzed sequences listed are accessible in publicly available databases.

## Supplementary Materials

The following supporting information can be downloaded at: https://www.mdpi.com/article/10.3390/antibiotics11121768/s1, Figure S1: Multiple sequence alignment of the newly identified *dfrB12–dfrB21*; Figure S2: Similarity and identity shared between *dfrB1–dfrB21*; Figure S3: Multiple sequence alignment of 20 newly identified *dfrB* homologues; Table S1: Prediction of chromosomal or plasmidic location; Table S2: Additional characteristics of the putative *dfrB* genes; Table S3: Genomic context analyses of *dfrB6*, *dfrB7*, *dfrB9* and *dfrB11* genes.

## References

[1] Huovinen P. (1987). Trimethoprim resistance. Antimicrob. Agents Chemother..

[2] Tjong E., Dimri M., Mohiuddin S.S. (2022). Biochemistry, Tetrahydrofolate. StatPearls.

[3] Fleming M.P., Datta N., Grüneberg R.N. (1972). Trimethoprim resistance determined by R factors. Br. Med. J..

[4] Howell E.E. (2005). Searching sequence space: Two different approaches to dihydrofolate reductase catalysis. Chembiochem.

[5] Eliopoulos G.M., Huovinen P. (2001). Resistance to Trimethoprim-Sulfamethoxazole. Clin. Infect. Dis..

[6] L’Abée-Lund T.M., Sørum H. (2001). Class 1 integrons mediate antibiotic resistance in the fish pathogen Aeromonas salmonicida worldwide. Microb. Drug Resist..

[7] Kadlec K., Kehrenberg C., Schwarz S. (2005). Molecular basis of resistance to trimethoprim, chloramphenicol and sulphonamides in Bordetella bronchiseptica. J. Antimicrob. Chemother..

[8] Tennstedt T., Szczepanowski R., Braun S., Pühler A., Schlüter A. (2003). Occurrence of integron-associated resistance gene cassettes located on antibiotic resistance plasmids isolated from a wastewater treatment plant. FEMS Microbiol. Ecol..

[9] Barlow R.S., Pemberton J.M., Desmarchelier P.M., Gobius K.S. (2004). Isolation and characterization of integron-containing bacteria without antibiotic selection. Antimicrob. Agents Chemother..

[10] Sunde M. (2005). Prevalence and characterization of class 1 and class 2 integrons in Escherichia coli isolated from meat and meat products of Norwegian origin. J. Antimicrob. Chemother..

[11] Toulouse J.L., Edens T.J., Alejaldre L., Manges A.R., Pelletier J.N. (2017). Integron-Associated DfrB4, a Previously Uncharacterized Member of the Trimethoprim-Resistant Dihydrofolate Reductase B Family, Is a Clinically Identified Emergent Source of Antibiotic Resistance. Antimicrob. Agents Chemother..

[12] Lemay-St-Denis C., Diwan S.-S., Pelletier J.N. (2021). The Bacterial Genomic Context of Highly Trimethoprim-Resistant DfrB Dihydrofolate Reductases Highlights an Emerging Threat to Public Health. Antibiotics.

[13] Toulouse J.L., Shi G., Lemay-St-Denis C., Ebert M.C.C.J.C., Deon D., Gagnon M., Ruediger E., Saint-Jacques K., Forge D., Vanden Eynde J.J. (2020). Dual-Target Inhibitors of the Folate Pathway Inhibit Intrinsically Trimethoprim-Resistant DfrB Dihydrofolate Reductases. ACS Med. Chem. Lett..

[14] Brisson N., Hohn T. (1984). Nucleotide sequence of the dihydrofolate-reductase gene borne by the plasmid R67 and conferring methotrexate resistance. Gene.

[15] Lemay-St-Denis C., Alejaldre L., Jemouai Z., Lafontaine K., St-Aubin M., Hitache K., Valikhani D., Weerasinghe N.W., Létourneau M., Thibodeaux C.J. (2022). A conserved SH3-like fold in diverse putative proteins tetramerises into an oxidoreductase providing an antimicrobial resistance phenotype. Philos. Trans. R. Soc. B Biol. Sci..

[16] Narayana N., Matthews D.A., Howell E.E., Nguyen-huu X. (1995). A plasmid-encoded dihydrofolate reductase from trimethoprim-resistant bacteria has a novel D2-symmetric active site. Nat. Struct. Biol..

[17] Krahn J.M., Jackson M.R., Derose E.F., Howell E.E., London R.E. (2007). Crystal Structure of a Type II Dihydrofolate Reductase Catalytic Ternary Complex. Biochemistry.

[18] West F.W., Seo H.-S., Bradrick T.D., Howell E.E. (2000). Effects of Single-Tryptophan Mutations on R67 Dihydrofolate Reductase. Biochemistry.

[19] Kneis D., Berendonk T.U., Forslund S.K., Hess S. (2022). Antibiotic Resistance Genes in River Biofilms: A Metagenomic Approach toward the Identification of Sources and Candidate Hosts. Environ. Sci. Technol..

[20] Szczepanowski R., Linke B., Krahn I., Gartemann K.H., Gützkow T., Eichler W., Pühler A., Schlüter A. (2009). Detection of 140 clinically relevant antibiotic-resistance genes in the plasmid metagenome of wastewater treatment plant bacteria showing reduced susceptibility to selected antibiotics. Microbiology.

[21] Hyatt D., Chen G.-L., LoCascio P.F., Land M.L., Larimer F.W., Hauser L.J. (2010). Prodigal: Prokaryotic gene recognition and translation initiation site identification. BMC Bioinform..

[22] Ebmeyer S., Kristiansson E., Larsson D.G.J. (2021). A framework for identifying the recent origins of mobile antibiotic resistance genes. Commun. Biol..

[23] Grigoriev I.V., Nordberg H., Shabalov I., Aerts A., Cantor M., Goodstein D., Kuo A., Minovitsky S., Nikitin R., Ohm R.A. (2012). The genome portal of the Department of Energy Joint Genome Institute. Nucleic Acids Res..

[24] Park H., Bradrick T.D., Howell E.E. (1997). A glutamine 67--> histidine mutation in homotetrameric R67 dihydrofolate reductase results in four mutations per single active site pore and causes substantial substrate and cofactor inhibition. Protein Eng..

[25] Martínez J.L., Coque T.M., Baquero F. (2015). Prioritizing risks of antibiotic resistance genes in all metagenomes. Nat. Rev. Microbiol..

[26] Pradier L., Tissot T., Fiston-Lavier A.S., Bedhomme S. (2021). PlasForest: A homology-based random forest classifier for plasmid detection in genomic datasets. BMC Bioinform..

[27] Krawczyk P.S., Lipinski L., Dziembowski A. (2018). PlasFlow: Predicting plasmid sequences in metagenomic data using genome signatures. Nucleic Acids Res..

[28] Néron B., Littner E., Haudiquet M., Perrin A., Cury J., Rocha E.P.C. (2022). IntegronFinder 2.0: Identification and Analysis of Integrons across Bacteria, with a Focus on Antibiotic Resistance in Klebsiella. Microorganisms.

[29] Siguier P., Perochon J., Lestrade L., Mahillon J., Chandler M. (2006). ISfinder: The reference centre for bacterial insertion sequences. Nucleic Acids Res..

[30] Cury J., Jové T., Touchon M., Néron B., Rocha E.P. (2016). Identification and analysis of integrons and cassette arrays in bacterial genomes. Nucleic Acids Res..

[31] McArthur A.G., Waglechner N., Nizam F., Yan A., Azad M.A., Baylay A.J., Bhullar K., Canova M.J., De Pascale G., Ejim L. (2013). The comprehensive antibiotic resistance database. Antimicrob. Agents Chemother..

[32] Karkman A., Do T.T., Walsh F., Virta M.P.J. (2018). Antibiotic-Resistance Genes in Waste Water. Trends Microbiol..

[33] Suzuki S., Pruden A., Virta M., Zhang T. (2017). Editorial: Antibiotic Resistance in Aquatic Systems. Front. Microbiol..

[34] Riesenfeld C.S., Goodman R.M., Handelsman J. (2004). Uncultured soil bacteria are a reservoir of new antibiotic resistance genes. Environ. Microbiol..

[35] Nikaido H. (1996). Multidrug efflux pumps of gram-negative bacteria. J. Bacteriol..

[36] Abella J., Fahy A., Duran R., Cagnon C. (2015). Integron diversity in bacterial communities of freshwater sediments at different contamination levels. FEMS Microbiol. Ecol..

[37] Ghaly T.M., Gillings M.R., Penesyan A., Qi Q., Rajabal V., Tetu S.G. (2021). The Natural History of Integrons. Microorganisms.

[38] Nguyen L.-T., Schmidt H.A., von Haeseler A., Minh B.Q. (2014). IQ-TREE: A Fast and Effective Stochastic Algorithm for Estimating Maximum-Likelihood Phylogenies. Mol. Biol. Evol..

[39] Imhoff J.F., Dworkin M., Falkow S., Rosenberg E., Schleifer K.-H., Stackebrandt E. (2006). The Phototrophic Beta-Proteobacteria. The Prokaryotes: Volume 5: Proteobacteria: Alpha and Beta Subclasses.

[40] Mistry J., Chuguransky S., Williams L., Qureshi M., Salazar G.A., Sonnhammer E.L.L., Tosatto S.C.E., Paladin L., Raj S., Richardson L.J. (2020). Pfam: The protein families database in 2021. Nucleic Acids Res..

[41] Fu L., Niu B., Zhu Z., Wu S., Li W. (2012). CD-HIT: Accelerated for clustering the next-generation sequencing data. Bioinformatics.

[42] Gobeil S.M.C., Gagné D., Doucet N., Pelletier J.N. (2016). ^15^N, ^13^C and ^1^H backbone resonance assignments of an artificially engineered TEM-1/PSE-4 class A β-lactamase chimera and its deconvoluted mutant. Biomol. NMR Assign..

[43] Studier F.W. (2005). Protein production by auto-induction in high density shaking cultures. Protein Expr. Purif..

[44] Alcock B.P., Raphenya A.R., Lau T.T.Y., Tsang K.K., Bouchard M., Edalatmand A., Huynh W., Nguyen A.V., Cheng A.A., Liu S. (2020). CARD 2020: Antibiotic resistome surveillance with the comprehensive antibiotic resistance database. Nucleic Acids Res..

[45] Katoh K., Standley D.M. (2013). MAFFT Multiple Sequence Alignment Software Version 7: Improvements in Performance and Usability. Mol. Biol. Evol..

[46] Letunic I., Bork P. (2021). Interactive Tree Of Life (iTOL) v5: An online tool for phylogenetic tree display and annotation. Nucleic Acids Res..

