# Supplementary material for: Discovery of Highly Trimethoprim-Resistant DfrB Dihydrofolate Reductases in Diverse Environmental Settings Suggests an Evolutionary Advantage Unrelated to Antibiotic Resistance
Source: Antibiotics (Basel). 2022 Dec 7;11(12):1768. doi: 10.3390/antibiotics11121768 (PMC9774602; doi:10.3390/antibiotics11121768)
Supplement: Supplementary file 1 [file antibiotics-11-01768-s001.zip › antibiotics-2082999-SI.pdf]

Supporting information

Discovery of highly trimethoprim resistant DfrB dihydrofolate reductases in diverse environmental settings suggests an evolutionary advantage unrelated to antibiotic resistance

Stella Cellier-Goetghebeur[1,2,3], Kiana Lafontaine[1,2,3], Claudèle Lemay-St-Denis[1,2,3], Princesse Tsamo[1,2,3], Alexis Bonneau-Burke[2,3,4], Janine N. Copp[5] and Joelle N. Pelletier[1,2,3,4,*]

[1] Department of Biochemistry and Molecular Medicine, Université de Montréal, Montréal, QC H3T 1J4, Canada
[2] PROTEO, The Québec Network for Research on Protein, Function, Engineering and Applications, Québec, QC G1V 0A6, Canada
[3] CGCC, Center in Green Chemistry and Catalysis, Montréal, QC H3A 0B8, Canada
[4] Chemistry Department, Université de Montréal, Montréal, QC H2V 0B3, Canada
[5] Michael Smith Laboratories, University of British Columbia, Vancouver, BC, Canada
[*] Correspondence: joelle.pelletier@umontreal.ca

Table of Contents:

Figure S1. Multiple sequence alignment of the newly identified *dfrB12-dfrB21.*
Figure S2. Similarity and identity shared between *dfrB1-dfrB21*.
Figure S3. Multiple sequence alignment of 20 newly identified *dfrB* homologues.
Table S1. Prediction of chromosomal or plasmidic location.
Table S2. Additional characteristics of the putative *dfrB* genes.
Table S3. Genomic context analyses of *dfrB6, dfrB7, dfrB9* and *dfrB11* genes.

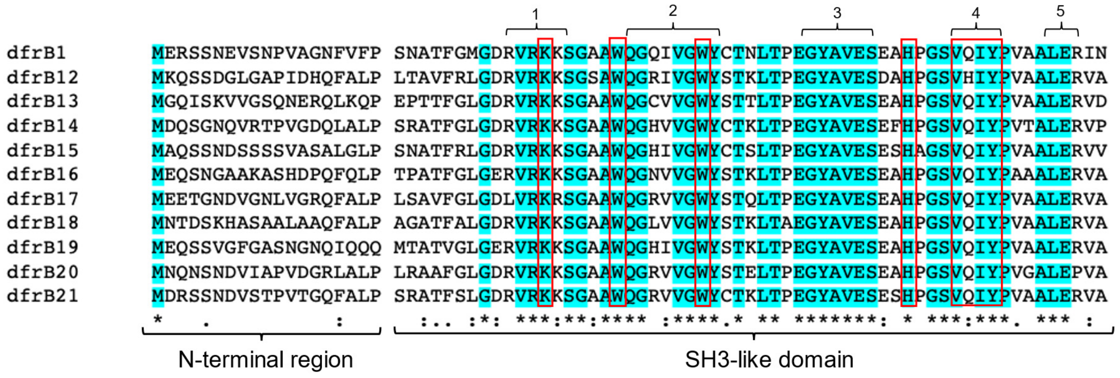

Figure S1. Multiple sequence alignment of the newly identified *dfrB12-dfrB21*. The *dfrB1* sequence serves as a reference. The 78 amino acid sequence comprises a poorly conserved, unstructured *N*-terminal region and a highly conserved region that encodes the SH3-like domain. The residues encoding the five β strands in the DfrB1 structure are identified with brackets and numbered. Amino acid conservation is indicated using standard annotation beneath the alignment and conserved residues are highlighted in cyan. Functionally and structurally important residues are framed in red. The alignment was produced using MAFFT.

	1	2	3	4	5	6	7	9	10	11	12	13	14	15	16	17	18	19	20	21
1		86	85	83	92	92	87	87	88	86	76	73	82	79	78	82	81	73	79	87
2	78		88	82	83	86	79	88	95	91	85	74	85	83	82	85	85	78	83	88
3	78	86		86	85	85	78	88	94	91	78	74	86	82	85	82	86	76	79	90
4	77	74	79		85	82	77	85	86	85	72	72	83	85	76	78	77	72	78	85
5	88	79	82	77		92	89	85	86	85	76	73	81	76	78	81	79	72	78	82
6	87	82	83	77	91		89	86	87	87	79	78	82	77	81	83	82	77	82	83
7	84	74	76	72	89	87		79	80	80	74	71	77	72	73	77	73	70	76	79
9	77	85	85	74	77	79	72		92	88	82	76	86	83	81	83	85	78	82	90
10	82	92	92	79	83	86	78	88		95	82	74	89	85	82	85	86	77	85	94
11	78	88	90	77	81	83	77	86	92		79	76	88	82	82	82	83	77	82	91
12	67	78	72	65	69	73	67	74	76	74		76	77	76	81	86	79	78	85	81
13	68	73	72	65	68	73	66	73	73	74	68		73	73	78	74	74	78	76	72
14	73	79	83	73	76	78	71	81	86	85	69	72		82	79	82	77	77	81	87
15	76	76	77	74	76	74	70	74	78	74	68	69	76		74	77	74	74	77	85
16	68	78	76	67	68	72	63	76	76	74	73	72	72	68		79	81	83	78	79
17	71	76	77	69	71	74	67	74	77	74	73	72	74	67	72		82	78	86	81
18	69	79	77	72	71	74	66	78	78	76	73	72	71	69	76	72		77	82	82
19	68	73	71	64	67	72	65	72	72	72	74	73	71	69	78	69	71		77	74
20	72	78	76	72	72	77	68	78	81	77	74	72	77	76	72	77	73	68		82
21	79	85	88	79	78	81	76	86	92	88	74	71	85	78	73	77	77	69	79	

Figure S2. Similarity (top right) and identity (bottom left) shared between *dfrB1-dfrB21*. The *dfrB* sequences are identified 1-21; there is no *dfrB8*. Values are percentages and are colored from a red (lowest sequence identity/similarity) to green gradient (highest sequence identity/similarity).

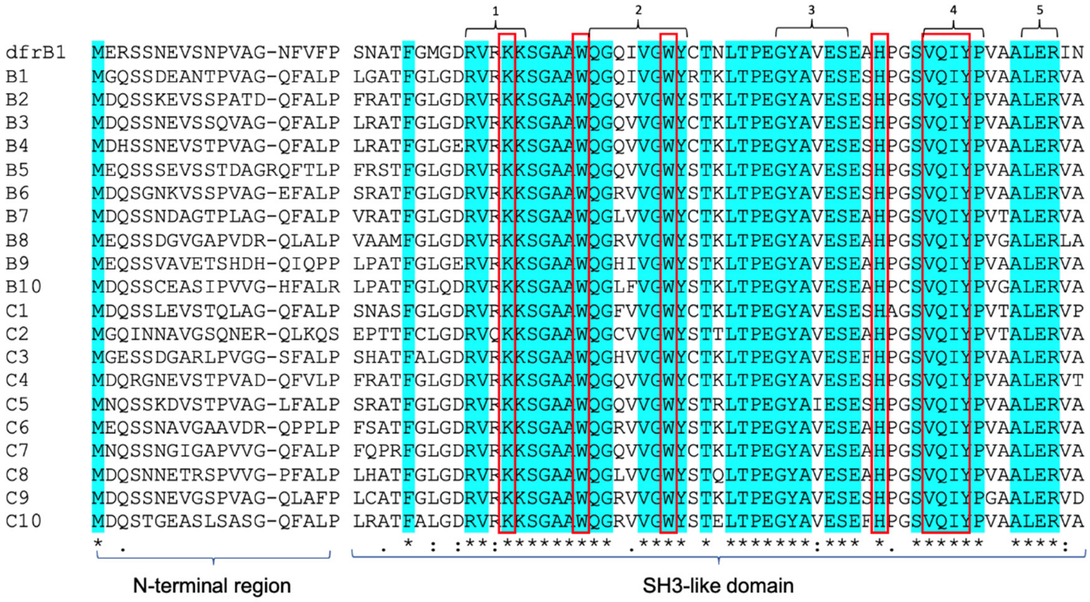

Figure S3. Multiple sequence alignment of 20 newly identified putative *dfrB* homologues. The *dfrB1* sequence serves as a reference. The 78 amino acid sequence comprises a poorly conserved, unstructured *N*-terminal region and a highly conserved region that encodes the SH3-like domain. The residues encoding the five β strands in the DfrB1 structure are identified with brackets and numbered. Amino acid conservation is indicated using standard annotation beneath the alignment and residues that are conserved between *dfrB1* and the newly identified putative *dfrB* are highlighted in cyan. Functionally and structurally important residues are framed in red. The alignment was produced using MAFFT.

Table S1. Prediction of chromosomal or plasmidic location.

Name	JGI/IMG identifier	PlasForest prediction [a]	PlasFlow prediction [b]
dfrB12	Ga0062593_101421357	Chromosome	Plasmid
dfrB13	Ga0062590_102580916	Chromosome	Plasmid
dfrB14	Ga0066412_1097643	Chromosome	Plasmid
dfrB15	Ga0075154_10665361	Chromosome	Plasmid
dfrB16	Ga0190269_11200979	Chromosome	Unclassified
dfrB17	Ga0193596_1008267	Chromosome	Unclassified
dfrB18	Ga0213876_11554563	Chromosome	Unclassified
dfrB19	Ga0311334_10409960	Chromosome	Unclassified
dfrB20	Ga0307508_10008142	Chromosome	Plasmid
dfrB21	Ga0394858_026559	Chromosome	Plasmid

[a] Contigs were classified as chromosomal or plasmidic using PlasForest.

[b] Contigs were classified as chromosomal or plasmidic using PlasFlow.

Table S2. Additional characteristics of the putative *dfrB* genes.

Name	GenBank / JGI ID [a]	Host Organism [a]
B1	MG649396	Uncultured bacterium
B2	AP024402	*Aeromonas caviae*, BPB
B3	JADMJN010000010	*Rhodoferax sp.*, BPB
B4	JABRWJ010000036	*Aquabacterium terrae*, BPB
B5	CP049957	*Bordetella trematum*, BPB
B6	JAFEDL010000101	Proteobacteria bacterium
B7	JACPOK010000019	*Burkholderiales* bacterium
B8	JADWYS010000001	*Caenimonas sp*. BPB
B9	JADKEH010000029	*Rhodoferax sp.*, BPB
B10	LMUW01000222	*Xenophilus sp.*, BPB
C1	Ga0214919_1000355031	*Comamonadaceae*, BPB
C2	Ga0075012_100199321	Undetermined
C3	Ga0335084_100502587	*Comamonadaceae*, BPB
C4	Ga0435267_00540993_3287_3523	*Comamonadaceae*, BPB
C5	Ga0394817_0033593_1221_1457	Proteobacteria
C6	JGI25613J43889_100117722	Proteobacteria
C7	Ga0239296_031039_1466_1702	Gammaproteobacteria
C8	Ga0105245_101602713	*Burkholderiales*
C9	Ga0307515_101793572	Proteobacteria
C10	rootL2_101999113	Undetermined

[a] Information was retrieved on JGI/IMG or NCBI databases for each sequence, if available.

Table S3. Genomic context analyses of *dfrB* genes.

Name and position [a]	Genomic context length (bp) [b]	Environment [c]	Species [d]	Integron [e]	Insertion sequences [f]	Organization [g]	Antibiotic resistance genes [h]
dfrB6 14723..14959	22326	Wastewater	*Salmonella enterica*, GPB	Complete (11572..17068)	Tn3 transposase (18634..19188), IS110 (19267..20271)	Plasmid	*BEL-1, CmlA, QacE, Sul1, GNAT*
dfrB7 1048..1296	2333	Salmon River, British Columbia	*Aeromonas hydrophila*, BPB	Complete (2..2250)	None	Chromosome	*aadA2*
dfrB9 4409..4645	40047	Clinical	*Enterobacteriaceae*, GPB	Complete (2149..9133)	Tn3 transposase (8670..10701)	Plasmid	*IMP-1, GNAT, AAC(6')-Ib4, Smr, CmlA, QacE, Sul1,*
dfrB11 114140..114376	114566	Groundwater (140-250m deep)	*Betaproteobacteria*	None	None	Chromosome	None

[a] Sequences were retrieved from NCBI.

[b] Number of base pairs (bp) in genomic context.

[c] Environmental source was determined using the available information on the NCBI database.

[d] Species information was retrieved from NCBI

[e] Complete and incomplete (CALIN) integrons within 5 kbp of a *dfrB* were searched for with IntegronFinder. Where applicable, the type of MGE identified and its position in the contig are indicated.

[f] Searched for with ISFinder, within 5 kbp of a *dfrB*.

[g] Organization as chromosomal or plasmidic was predicted with PlasForest using DNA contig sequences.

[h] Searched for on the CARD database, within 5 kbp of a *dfrB*.
